# Bio-inspired neural networks for decision-making mechanisms and neuromodulation for motor control in a differential robot

**DOI:** 10.3389/fnbot.2023.1078074

**Published:** 2023-02-03

**Authors:** Roberto Jose Guerrero-Criollo, Jason Alejandro Castaño-López, Julián Hurtado-López, David Fernando Ramirez-Moreno

**Affiliations:** ^1^Department of Engineering, Universidad Autónoma de Occidente, Cali, Colombia; ^2^Department of Mathematics, Universidad Autónoma de Occidente, Cali, Colombia; ^3^Department of Physics, Universidad Autónoma de Occidente, Cali, Colombia

**Keywords:** bio-inspired neural network, neuromodulation network, adaptation stage, signal processing, differential robot, exploration behavior, automaton

## Abstract

The aim of this work is to propose bio-inspired neural networks for decision-making mechanisms and modulation of motor control of an automaton. In this work, we have adapted and applied cortical synaptic circuits, such as short-term memory circuits, winner-take-all (WTA) class competitive neural networks, modulation neural networks, and nonlinear oscillation circuits, in order to make the automaton able to avoid obstacles and explore simulated and real environments. The performance achieved by using biologically inspired neural networks to solve the task at hand is similar to that of several works mentioned in the specialized literature. Furthermore, this work contributed to bridging the fields of computational neuroscience and robotics.

## 1. Introduction

Decision-making is a process in animals that allow them to increase their chances of survival. Decision-making includes, for example, knowing when to flee from a threat, avoiding the consuming of spoiled food, or even performing attacking or breeding behaviors. Understanding how decision-making mechanisms work within the cerebral cortex and generating a model of their output behaviors has been a focus of research in neuroscience. In Hurtado-López et al. (2017) and Hurtado-López and Ramirez-Moreno (2019) authors describe the model of a neural network that mimics social behavior in mice involving breeding and attack interactions. As mentioned in Hikosaka et al. (2018) the basal ganglia control body movements. In addition, it is involved in behavioral changes in animals. Héricé et al. (2016) propose a neural network model of the basal ganglia based on spiking neurons. The developed model allows second-level decision-making to be performed as in primates. There are other experiments, performed on Drosophila flies and consisting of introducing them into a flight simulator containing green and blue colored regions. If the fly stood on the blue regions it received a heat punishment. The results gave evidence that these insects have the ability to adjust their flight behavior based on visual color information. In Wei et al. (2017) a model based on Spiking Neural Networks (SNN) and postsynaptic plasticity is proposed to describe in a mathematical way both the decision-making behavior based on visual information received by Drosophila and the learning process.

Providing robotic navigation systems with the capacities mentioned above is of great interest in order to enhance their efficiency and autonomy. Zhao et al. (2020) developed an SNN model that allows describing the experimental behavior in Drosophila and implementing it in a UAV (Unmanned Aerial Vehicle). The results show that with the proposed model the UAV learns to make decisions quickly from the available visual information similar to the experiment. The closest approach to ours is made by Pardo-Cabrera et al. (2022), in which a bio-inspired navigation and exploration system for a robotic hexapod is developed. In this work, a network of social behaviors in mice, proposed in Hurtado-López et al. (2017) and Hurtado-López and Ramirez-Moreno (2019) is modified to perform homing, exploration, and approaching behaviors in robots. We propose a decision network to perform exploration in robots, and present as a novelty the implementation of a network inspired by the basal ganglia proposed by Ramirez-Moreno and Sejnowski (2012) to moderate the decision taken by the main network, reducing the reactivity of the system and providing greater safety in the navigation of the mobile platform.

Using bio-inspired neural networks allows us to perform numerous adaptations from animal-human behaviors and kinetics into autonomous robots. For instance, the performance of fast learning mechanisms for continuous adaptation or flexible plasticity in sensory pathways, in order to generate stable self-organized locomotion, deals with failures and adaptions to different walking in robots. In addition, it is clear how bio-inspired networks can work combined with distributed neural CPG, proprioceptive sensory adaptation, and body-environment interaction, achieving adaptive and flexible interlimb coordination for walking robots, as mentioned in Miguel-Blanco and Manoonpong (2020).

Additionally, the use of frequency is useful in order to control the locomotion of an automaton. Previous results show that the integration between motor pattern mechanisms and adaptation with a CPG-RBF leads to locomotion control of a hexapod robot in a complex environment. This kind of frequency adaptation not only significantly reduces energy use but also is comparable to the biological behaviors observed in animal locomotion (Thor et al., 2021).

As we have seen in previous works, there are numerous architectures of bio-inspired neural networks for the motor control of robots and automata. In this work, a novel bio-inspired neural network was designed for the control of the right and left actuators of a differential robot. For the latter, a reciprocal lateral inhibition circuit was used, which projects periodic cyclic signals and generates antagonistic nonlinear oscillations. The neuronal activity of these synaptic circuits with reciprocal lateral inhibition is typical of the motor control systems of periodic tasks such as breathing, swimming, or walking in vertebrates, among others.

Other approaches bring us a neat use of CPGs in order to control a sprawling quadruped robot (Suzuki et al., 2021), contributing to decentralized control with cross-couple sensory feedback to shaping body-limb coordination, which differs from previous research based on CPGs that works with inter-oscillator couplings or gait patterns based on geometric mechanics.

Ngamkajornwiwat et al. (2020) propose an online self-adaptive locomotion control technique based on the integration of a modular neural locomotion control (MNCL) and an artificial hormone mechanism (AHM) for a walking hexapod robot. Their contribution allows robot control without needing its kinematics, environmental model, and exteroceptive sensors. The technique performed relies only on a correlation between a predicted foot contact signal and the incoming foot contact signal from proprioceptive sensors. The steering and velocity regulation of the robot is achieved.

Most recent research introduces two new concepts in order to develop bio-inspired neural networks for the motor control of an automaton, the Self-Organizing Map (SOM) and the Spiking Neural Networks (SNN). Zahra et al. (2022) integrate both architectures, the SNN in a motor cortex-like differential map transforming motor plans from task-space to joint-space motor commands, and the SOM in a static map correlating joint-spaces of the robot and a teaching agent, which allows a robotic arm to learn from human actions, thus, the robotic arm learns by imitation.

Spiking Neural Network Models are based on the action potential firing temporal sequences. Usually, the leaky integrate-and-fire (LIF) neuron model is used in these networks. This model involves biophysics properties of the neuron such as membrane capacitance, conductance, and resting potential.

Current research shows wide implementations of SNN in mobile automata's navigation tasks. In Cao et al. (2015) a three-layer SNN-based controller is designed and implemented for target tracking of a mobile robot. Environmental information and target information are provided by CCD cameras, encoders, and ultrasonic sensors. The authors implemented a learning strategy based on Hebb's rule to modify the synaptic weights in the connections of the neural network (NN) in charge of the tracking task. Besides, the synaptic weights from a NN specialized in obstacle avoidance are defined by the designer and do not change in time. This strategy seeks to have more relevance in the obstacle avoidance task than that in the tracking task.

Shim and Li (2017), Lobov et al. (2020), and Liu et al. (2022) addressed the use of SNN for the motor control of mobile robots. Liu et al. (2022) proposed a biological autonomous learning algorithm based on reward modulated spike-timing-dependent plasticity (STDP). Taking this into consideration, an automaton can improve its decision-making in obstacle avoidance by a few sessions of trial-and-error in presence of new environments providing robustness to the exploration task. Approaching the cognitive and perception functions instrumented in automata behaviors, Macktoobian and Khataminejad (2016) developed a high-level cognitive behavior into a reactive agent, a Braitenberg vehicle (BV). Low-level perception is obtained by an SNN-Curved trajectory detection (CDT) model with which the motion of an agent in the environment is detected. The vehicle's control for producing the desired behaviors depending on the perception is made by an engineering method, approaching and fleeing behaviors are obtained.

Neurons respond to stimuli by generating action potentials. To describe the state of a neuron, the mean firing rate (MFR) of these action potentials can be taken. The dynamics of the NN based on MFR models can bring a better understanding of the expected behavior of the neuron at first sight than in SNN. Architectures based on SNN found in the literature have solved decision-making and motor control tasks. As mentioned in Suzuki et al. (2021), Thor et al. (2021), and Pardo-Cabrera et al. (2022), MFR models have been implemented to solve these tasks as well. The literature reviewed in this work shows that MFR models which satisfy the decision-making and control-motor for mobile automata navigation have not been explored widely. The present work proposes the design and implementation of an automaton's bio-inspired navigation framework using a mathematical MFR neural model described by Wilson and Cowan (1972).

SNN models have an advantage in single event-based learning on Hebb's rule. In our work, the advantage offered by MFR models is that it simply assimilates the advantage given in SNN models for single-event-based learning. We have achieved this in the meta-control network. Single-event-based learning enables modifying the performance of the automaton by setting the network's parameters in a single trial. In this work, the network's parameters are not modified, and for this reason, it cannot be considered a learning mechanism. The improvement in performance is obtained through the meta-control network. This network allows the adaptation of the automaton's behavior to significant environment changes (pop-up novelties) and dynamic obstacles, this is obtained by properly modulating the velocity applied to the robot's wheels. For the proposed network, the experimentation results show an improvement in the obstacle avoidance task when the meta-control network is involved. The literature consulted shows a recurrence of SNN models rather than mean firing rate models at the cost of the loss of a certain mathematical simplicity. As already mentioned, our model advantage is the combination of such mathematical simplicity and the formulation of single event-based learning.

## 2. Materials and methods

This section will explain the implemented bio-inspired neural circuits, the neural network design itself, the adaptation stage, and the signal processing.

### 2.1. Bio-inspired neural network design

**Figure 1:**
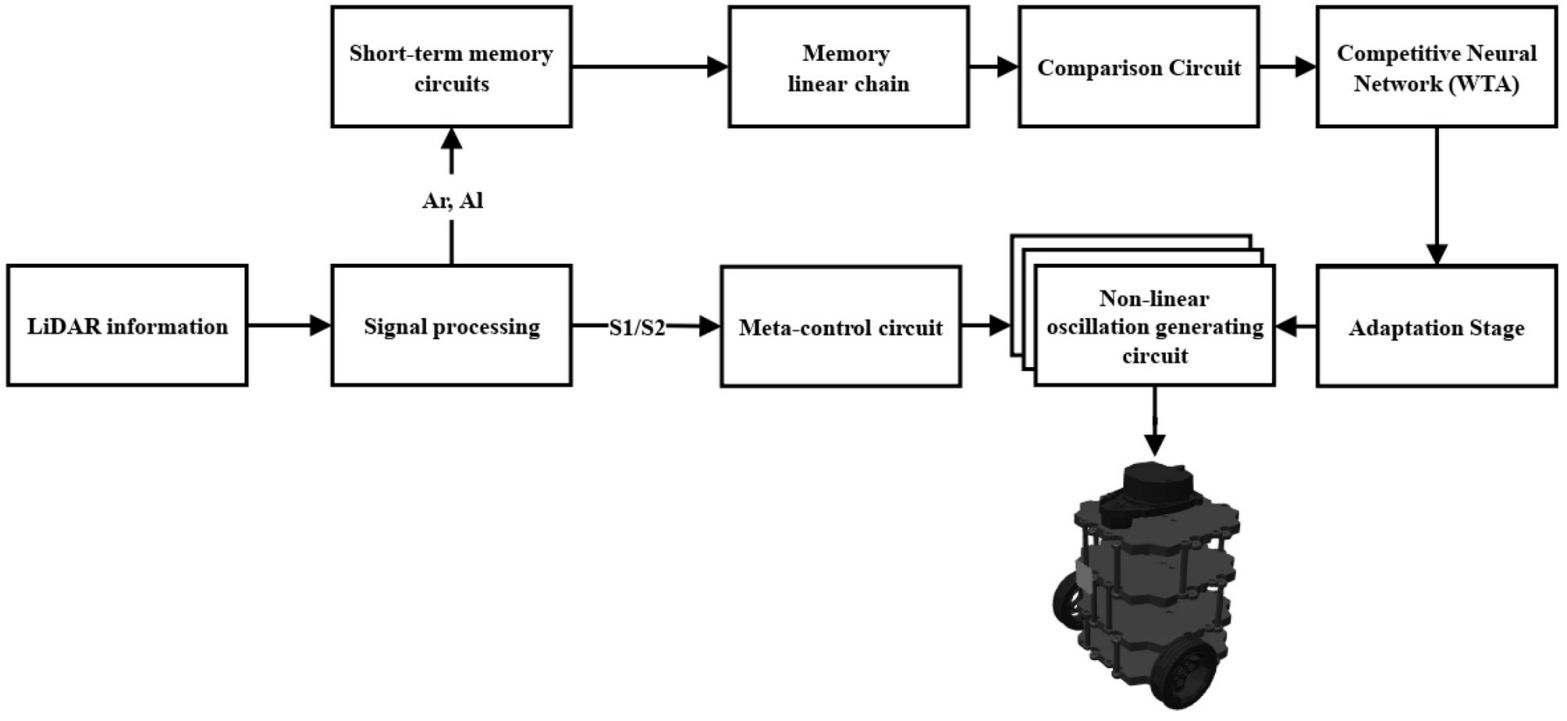
Architecture of the bio-inspired network for the exploration behavior.

As seen in Figure 1, the information perceived from the environment is captured by a LiDAR sensor and is processed by the *Signal processing* block (Section 2.5). From this block, the signals *A*_*r*_ and *A*_*l*_, and *S*_1_ and *S*_2_ are processed. The *A*_*r*_ and *A*_*l*_ signals convey information about the obstacle's presence or absence in the right and left areas, respectively. These signals enter the *Short-term memory circuits* block (Section 2.2.1) which extends their information in time. The projections from the previous block enter the *Memory linear chain* block (Section 2.2.2) and retain and increase the intensity of the projections. In the *Comparison Circuit* block (Section 2.2.3) the projections from the Memory linear chain block are compared and thus promote a faster decision by the Competitive Neural Network (WTA) block (Section 2.2.4). In the Competitive Neural Network (WTA) block, the projections of the previous block are compared, and a proper motion decision is obtained among rightward, leftward, and forward. With the *Adaptation Stage* block (Section 2.2.5) it is possible to detect a tendency among the motions that have been executed in a time interval and thus adapt the parameters of the *Non-linear oscillation generating circuit* block (Section 2.2.7). The *Non-linear oscillation generating circuit* block, produces the signals for the automaton motor execution. Finally, the *Meta-control circuit* block (Section 2.2.6) modulates the rightward, leftward, and forward movements, allowing an improvement of the performance in situations where a novelty is prioritized before a previously weighted decision. The *Meta-control circuit* block is fed by the *S*_1_ signal, which corresponds to the information of any new obstacle, and by forced complementarity, we obtain the *S*_2_ signal. Forced complementary is understood as a decremental response to an incremental stimulus, obtained by the substruction between a threshold and the stimulus.

The neuron model used in this work takes inspiration from the basic negative feedback loop described by Wilson and Cowan (1972), in which connections with arrow endings represent excitatory projections and connections with circled endings represent inhibitory projections.

The response (*R*) of a neuron to a single stimulus (*P*) is described by the differential (Equation 1) (Wilson and Cowan, 1972) where τ is the time constant.


(1) $$\begin{array}{l} \frac{dR}{dt}=\frac{1}{\tau }\left(-R+\text{Ψ}\left(M,P,\sigma \right)\right) \end{array}$$

Ψ(*M, P*, σ) is the Naka-Rushton activation function (Wilson and Cowan, 1972), and is implemented as a mathematical approximation of these responses. *M* is the maximum firing rate for a very intense stimulus and σ, called the half-saturation constant, determines the value at which Ψ(*M, P*, σ) reaches half of its maximum. The mathematical representation is given in Equation (2).


(2) $$\begin{array}{l} \Psi (M,P,\sigma )=\{\begin{array}{ll} \frac{MP^{2}}{\sigma^{2}+P^{2}} & \text{for }P\geq 0\\ 0 & \text{for }P<0 \end{array} \end{array}$$

### 2.2. Cortical synaptic circuits

The bio-inspired neural networks implemented in this work are based on cortical synaptic circuits (Ramirez-Moreno and Hurtado-Lopez, 2014). These circuits are observed in the cerebral cortex, subcortical nuclei, and in the spinal cord.

Next, we present some cortical synaptic circuits which provide good performance in our robot framework. The structure of the differential equations of these circuits corresponds to that shown in Equation (1) and the parameters used are summarized in Table 1. These parameters were obtained by the heuristic method, some of them were taken from Guerrero-Criollo et al. (2022).

**Table 1:** Parameters.

**Parameters**	**Value**
*A*	100
*A*_*cn*_	1
*B*	125
*C*	30
*D*	900
*B*_*cn*_	1
*N*_*cn*_	40
*M*_*cn*_	0.3
*a*	3
*b*	0.7
ϕ	1
*c*	1
β	1.5
θ	1
*g*	1
*h*	1
*d*	3.2
Θ	10
τ	100 ms
τ_ϵ_	1 ms
τ_1_	20 ms
τ_2_	2,000 ms
τ_6_	10 ms
τ_4_	10 ms
τ_5_	5,000 ms
τ_7_	20 ms
τ_8_	280 ms
*Oe*	1
ϵ	0.5
γ	100
ω	50
Ω	5
δ	300
ρ	11
ψ	0.006
*f*(*x*)	$\left\{ \begin{array}{cc} 1 & \text{for}x\geq 0.3\\ 0 & \text{otherwise} \end{array}\right.$

#### 2.2.1. Short-term memory circuits. Recurrent excitatory

Recurrent excitatory circuits within the Central Nervous System (CNS) allow short-term retention of information. The function of the adapting interneurons is to execute a delayed control task over the main units.


(3) $$\begin{array}{l} \frac{dZ_{1}}{dt}=\frac{1}{\tau_{1}}\left(-Z_{1}+\text{Ψ}\left(A,\;\omega A_{r}+aZ_{3},\;B+cZ_{2}\right)\right) \end{array}$$

(4) $$\begin{array}{l} \frac{dZ_{2}}{dt}=\frac{1}{\tau_{2}}\left(-Z_{2}+bZ_{1}\right) \end{array}$$

(5) $$\begin{array}{l} \frac{dZ_{3}}{dt}=\frac{1}{\tau_{1}}\left(-Z_{3}+\text{Ψ}\left(A,\;aZ_{1},\;B+cZ_{4}\right)\right) \end{array}$$

(6) $$\begin{array}{l} \frac{dZ_{4}}{dt}=\frac{1}{\tau_{2}}\left(-Z_{4}+bZ_{3}\right) \end{array}$$

(7) $$\begin{array}{l} \frac{dY_{1}}{dt}=\frac{1}{\tau_{1}}\left(-Y_{1}+\text{Ψ}\left(A,\;\omega A_{l}+aY_{3},\;B+cY_{2}\right)\right) \end{array}$$

(8) $$\begin{array}{l} \frac{dY_{2}}{dt}=\frac{1}{\tau_{2}}\left(-Y_{2}+bY_{1}\right) \end{array}$$

(9) $$\begin{array}{l} \frac{dY_{3}}{dt}=\frac{1}{\tau_{1}}\left(-Y_{3}+\text{Ψ}\left(A,\;aY_{1},\;B+cY_{4}\right)\right) \end{array}$$

(10) $$\begin{array}{l} \frac{dY_{4}}{dt}=\frac{1}{\tau_{2}}\left(-Y_{4}+bY_{3}\right) \end{array}$$

**Figure 2:**
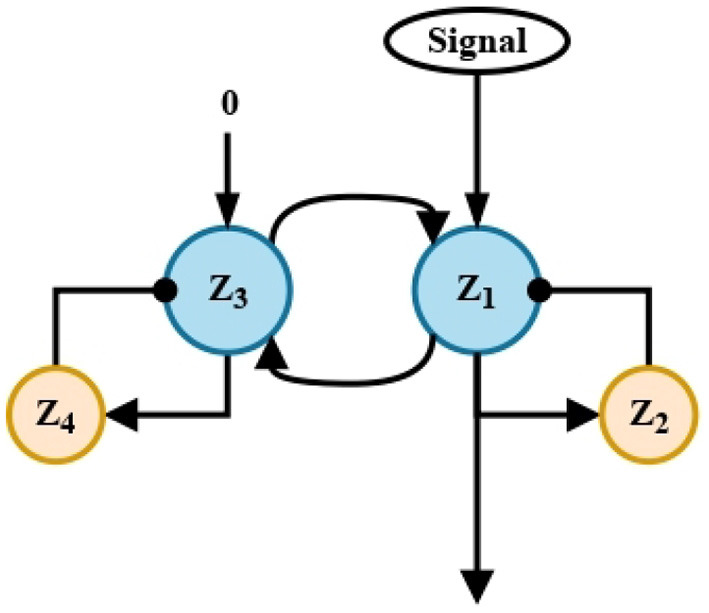
Short-term memory circuit. Recurrent excitation. Two circuits similar to that shown above, process the signals *A*_*r*_ and *A*_*l*_, respectively. The four processing units shown correspond to the units named in Equations (3)–(6) y (7)–(10).

The purpose of this short-term memory circuit is to extend in time the information captured from the environment, *A*_*r*_ and *A*_*l*_. For this purpose, each signal enters through a single channel to the recurrent excitation circuit, where the main neurons *Z*_1_ and *Y*_1_, respectively, process the signals. Input signals are maintained by the delayed feedback of neurons *Z*_2_ and *Y*_2_, as shown in Figure 2.

These circuits replicate those found in the CNS associated with the short-term memory mechanism that, for instance, serves to recall a phone number for immediate use. Without them, the automaton, like its biological counterpart, would not be able to remember the previously processed information itself, in this case, it would not be able to remember whether there was an obstacle before or not.

#### 2.2.2. Memory linear chain

**Figure 3:**
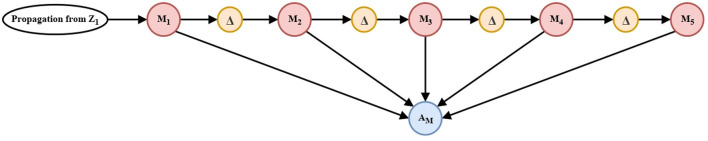
Memory linear chain. Two circuits similar to that shown above, process the propagation of the short-term memory circuit (Figure 2). The five processing units shown correspond to the units named in Equations (11), (12) y (14), (15).

Projections from recurrent excitatory circuits (Figure 2) enter a memory linear chain of five neurons (Figure 3).


(11) $$\begin{array}{l} \frac{dM_{1}}{dt}=\frac{1}{\tau }\left(-M_{1}+\text{Ψ}\left(A,Z_{1},C\right)\right) \end{array}$$

(12) $$\begin{array}{l} \frac{dM_{i}}{dt}=\frac{1}{\tau }\left(-M_{i}+\text{Ψ}\left(A,\;M_{\left(i-1\right)},\;C\right)\right),\;i=2,3,\ldots ,5. \end{array}$$

(13) $$\begin{array}{l} \frac{dA_{M}}{dt}=\frac{1}{\tau_{\epsilon }}\left(-A_{M}+\text{Ψ}\left(A,\;\overset{5}{\underset{i=1}{\sum }}M_{i},\;C\right)\right) \end{array}$$

(14) $$\begin{array}{l} \frac{dN_{1}}{dt}=\frac{1}{\tau }\left(-N_{1}+\text{Ψ}\left(A,\;Y_{1},\;C\right)\right) \end{array}$$

(15) $$\begin{array}{l} \frac{dN_{i}}{dt}=\frac{1}{\tau }\left(-N_{i}+\text{Ψ}\left(A,\;N_{\left(i-1\right)},\;C\right)\right),\;i=2,3,\ldots ,5. \end{array}$$

(16) $$\begin{array}{l} \frac{dA_{N}}{dt}=\frac{1}{\tau_{\epsilon }}\left(-A_{N}+\text{Ψ}\left(A,\;\overset{5}{\underset{i=1}{\sum }}N_{i},\;C\right)\right) \end{array}$$

Implementation of the memory linear chain above seeks to retain and enhance the intensity of the projections. Each memory unit is separated from each other by delay units (Δ*t*) and their projections are accumulated in the accumulation neurons *A*_*M*_ (Equation 13) and *A*_*N*_ (Equation 16), respectively, for each processing channel, Figure 3.

Putting together the memory linear chain and the short-term memory circuits, it is possible to obtain an approximation to working memory. And thus, like its biological counterpart, the automaton could retain the previously processed information.

#### 2.2.3. Comparison circuit

**Figure 4:**
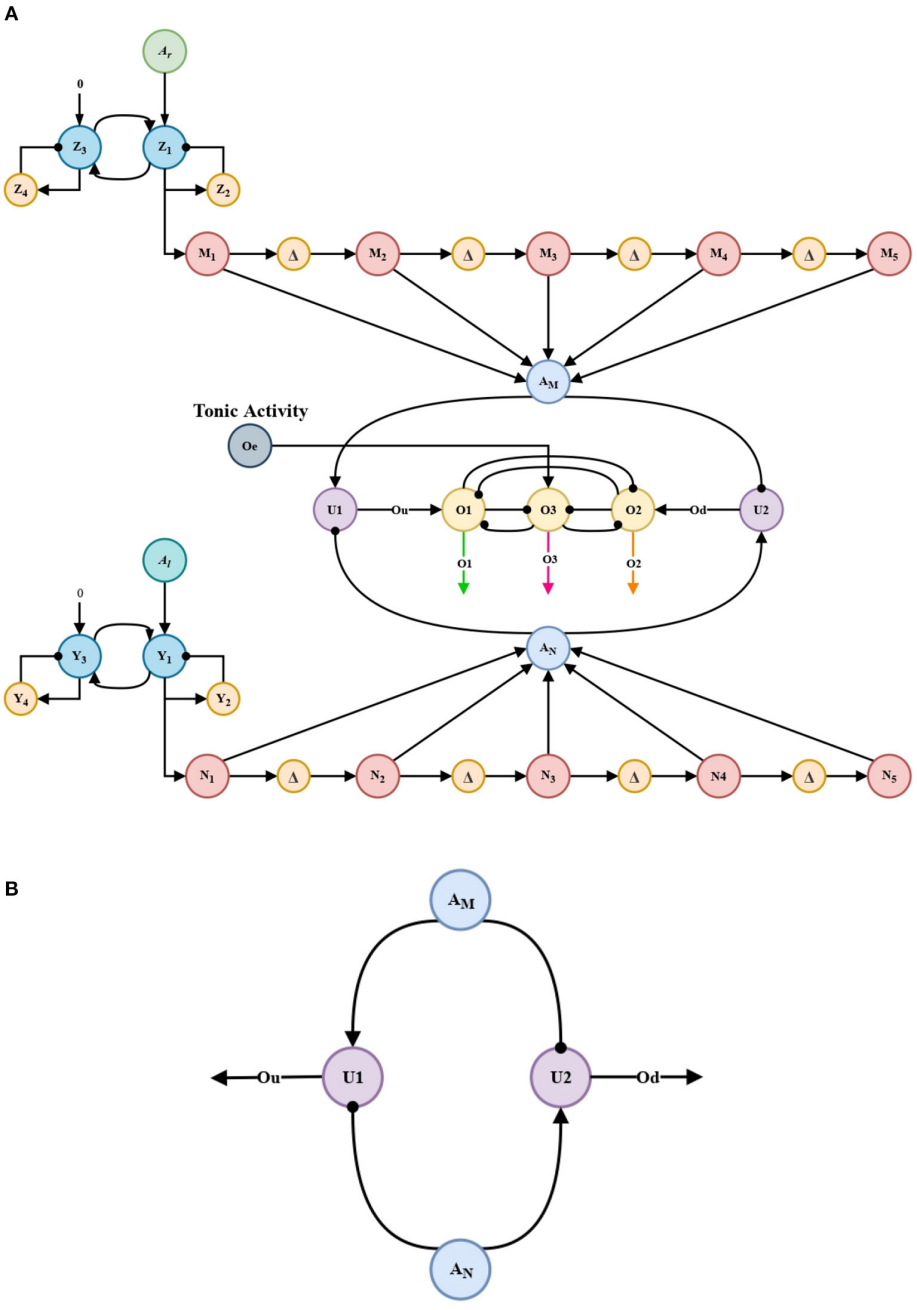
Architecture of the bio-inspired network for the exploration behavior. **(A)** Architecture of the bio-inspired network for the exploration behavior. **(B)** comparison circuit.

Units *U*_1_ and *U*_2_ in Figure 4B act as comparison neurons. These units receive the reciprocal excitatory and inhibitory projections from the accumulation neurons *A*_*M*_ and *A*_*N*_, see Figure 4.


(17) $$\begin{array}{l} \frac{dU_{1}}{dt}=\frac{1}{\tau_{\epsilon }}\left(-U_{1}+\frac{max\left(0,\;A\left(A_{M}-A_{N}\right)\right)}{D+A_{M}-A_{N}}\right) \end{array}$$

(18) $$\begin{array}{l} \frac{dU_{2}}{dt}=\frac{1}{\tau_{\epsilon }}\left(-U_{2}+\frac{max\left(0,\;A\left(A_{N}-A_{M}\right)\right)}{D+A_{N}-A_{M}}\right) \end{array}$$

Projections from accumulation neurons *A*_*M*_ and *A*_*N*_ are compared in order to promote a faster and clearer decision by the WTA (winner-take-all) decision circuit. *U*_1_ and *U*_2_ units from Equations (17), (18) produce the *O*_*u*_ and *O*_*d*_ signals, which together with a tonic activity *O*_*e*_, feeds the WTA circuit. This allows to know which neuron (*A*_*M*_ or *A*_*N*_) has an advantage in decision-making.

These circuits are based on a circuit present in the orbito-frontal cortex in primates. Without them, the automaton, like its biological counterpart, would not be able to take decisions, in this case, would not be able to take a decision between each processing channel (*A*_*M*_ or *A*_*N*_).

#### 2.2.4. Competitive neural networks. Decision circuit

Winner-take-all (WTA) decision circuits are neural networks that identify and choose the strongest input stimulus presented to the neural network. The decision-making mechanisms presented are inspired by those found in the Central Nervous System in primates.

In mammals and complex animals, we observe behaviors that come from decisions made in the face of different options, decisions that bring short- or long-term gains or benefits. For example, searching for food in the presence of predators, fleeing or fighting in threatening situations, or mating competition. The same applies to a mobile automaton that makes decisions based on stimuli taken from the environment, for instance, the slope of a terrain, its humidity, radioactivity, and viscosity, among others (Guerrero-Criollo et al., 2022).

**Figure 5:**
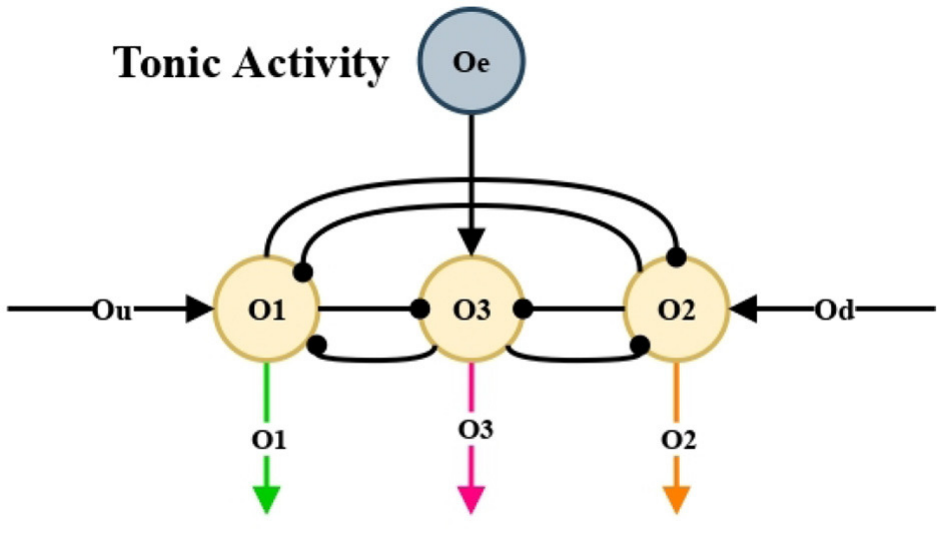
Competitive neural network. Class winner-take-all (WTA).

Ramirez-Moreno and Hurtado-Lopez (2014) proposed a basic neural network that chooses an option between two alternatives presented. In this work, that network was modified in order to make a selection among three alternatives, see Figure 5.


(19) $$\begin{array}{l} \frac{dO_{1}}{dt}=\frac{1}{\tau }\left(-O_{1}+\text{Ψ}\left(\Omega A,\;U_{1}-a\left(O_{2}+O_{3}\right),\;B\right)\right) \end{array}$$

(20) $$\begin{array}{l} \frac{dO_{2}}{dt}=\frac{1}{\tau }\left(-O_{2}+\text{Ψ}\left(A,\;U_{2}-a\left(O_{1}+O_{3}\right),\;B\right)\right) \end{array}$$

(21) $$\begin{array}{l} \frac{dO_{3}}{dt}=\frac{1}{\tau }\left(-O_{3}+\text{Ψ}\left(A,\;O_{e}-a\left(O_{1}+O_{2}\right),\;B\right)\right) \end{array}$$

Our aim is to make units *O*_1_, *O*_2_ and *O*_3_ to compete and reach a decision among leftward, rightward, and forward movements, see Figure 5. These signals *O*_1_, *O*_2_, and *O*_3_ are passed to the nonlinear oscillation generator circuits, individually, and in this way generate the motor control of the mobile automaton.

#### 2.2.5. Adaptation stage

The memory linear chain with delay units (Δ*t*) feeds the accumulation neurons *A*_*P*_ and *A*_*Q*_ Equation (24), respectively.


(22) $$\begin{array}{l} \frac{dP_{1}}{dt}=\frac{1}{\tau }\left(-P_{1}+\text{Ψ}\left(A,\;\alpha O_{1},\;C\right)\right) \end{array}$$

(23) $$\begin{array}{l} \frac{dP_{i}}{dt}=\frac{1}{\tau }\left(-P_{i}+\text{Ψ}\left(A,\;P_{\left(i-1\right)},\;C\right)\right),\;i=2,3,\ldots ,5. \end{array}$$

(24) $$\begin{array}{l} \frac{dA_{P}}{dt}=\frac{1}{\tau }\left(-A_{P}+\text{Ψ}\left(A,\;\overset{5}{\underset{i=1}{\sum }}P_{i},\;C\right)\right) \end{array}$$

**Figure 6:**
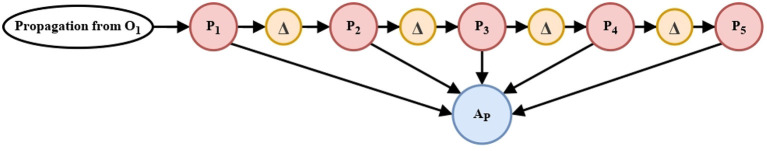
Memory linear chain for the adaptation stage. Two circuits similar to that shown above, process the propagation of *O*_1_ and *O*_2_ in the meta-decision circuit (Figure 5). The five processing units shown correspond to the units named in Equations (22)–(24).

Our aim is to have the behaviors tendency in a period of time and thus adapt the parameters in the nonlinear oscillation generator circuits, see in Figure 6.

Similar to the Memory linear chain (Section 2.2.2), this circuit oversees retaining the previously processed information in a time interval, and without it, the automaton would not be able to adapt its parameters in order to prioritize one of its three behaviors.

#### 2.2.6. Meta-control circuit

**Figure 7:**
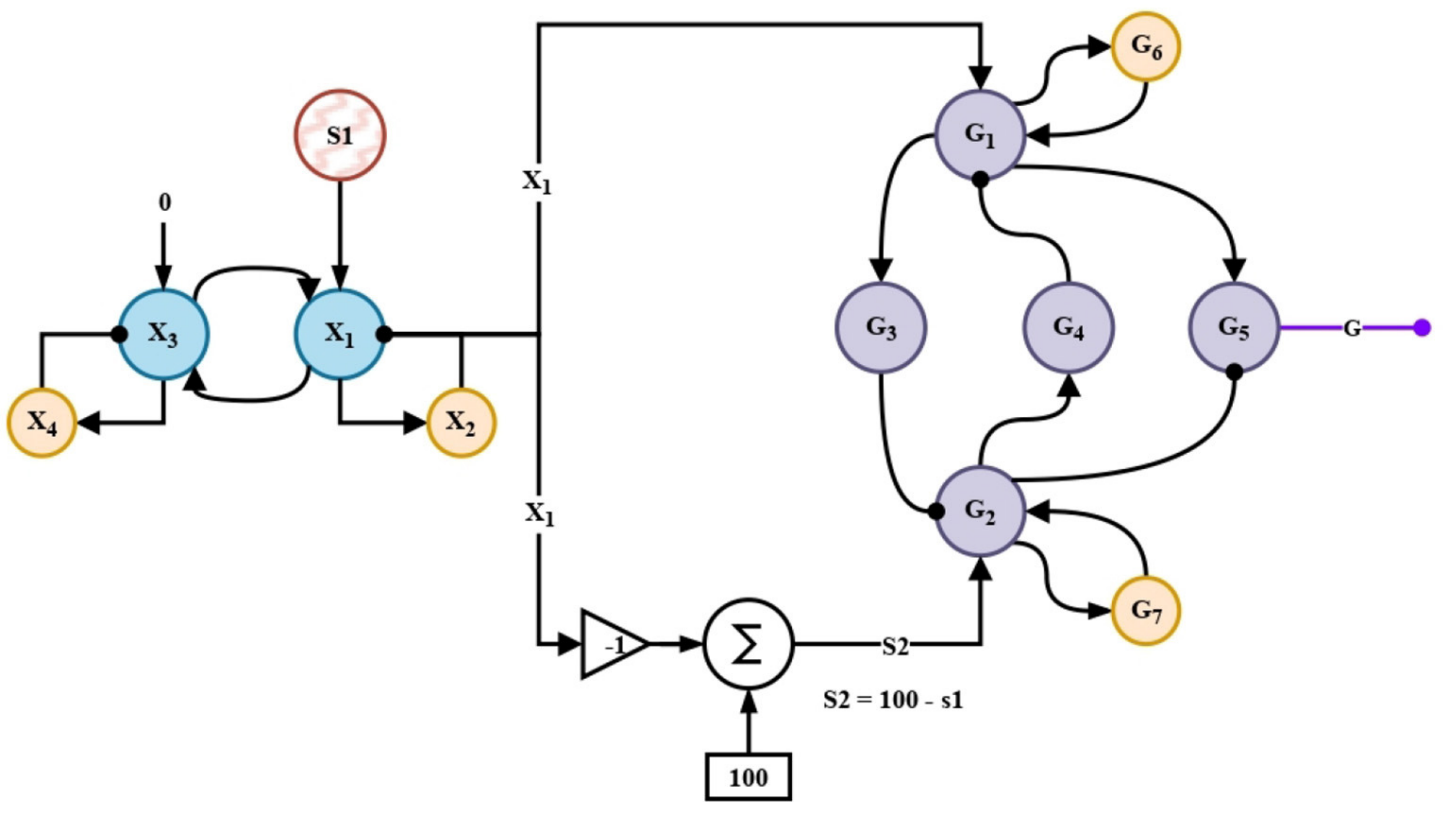
Meta-control circuit.

The CNS has neuromodulators in charge of transforming or changing the result of a primary operation under a pop-up novelty. In this work for the processing of this novelty, the modulation neural network described in Ramirez-Moreno and Sejnowski (2012) was implemented. Unit *G*_5_ in Figure 7 projects an inhibition signal to the motor behaviors. The modulation of these behaviors allows a better performance that mimics the reactions of complex animals in situations where a novelty is prioritized before a previously weighted decision.


(25) $$\begin{array}{l} \frac{dX_{1}}{dt}=\frac{1}{\tau_{4}}\left(-X_{1}+\text{Ψ}\left(A,\;\omega S_{1}+aX_{3},\;B+cX_{2}\right)\right) \end{array}$$

(26) $$\begin{array}{l} \frac{dX_{2}}{dt}=\frac{1}{\tau_{5}}\left(-X_{2}+bX_{1}\right) \end{array}$$

(27) $$\begin{array}{l} \frac{dX_{3}}{dt}=\frac{1}{\tau_{4}}\left(-X_{3}+\text{Ψ}\left(A,\;aX_{1},\;B+cX_{4}\right)\right) \end{array}$$

(28) $$\begin{array}{l} \frac{dX_{4}}{dt}=\frac{1}{\tau_{5}}\left(-X_{4}+bX_{3}\right) \end{array}$$

(29) $$\begin{array}{l} \frac{dG_{1}}{dt}=\frac{1}{\tau_{6}}\left(-G_{1}+\text{Ψ}\left(A_{cn},\;X_{1}-\phi G_{4}+G_{6},\;N_{cn}\right)\right) \end{array}$$

(30) $$\begin{array}{l} \frac{dG_{2}}{dt}=\frac{1}{\tau_{6}}\left(-G_{2}+\text{Ψ}\left(A_{cn},\;S2-\theta G_{3}+G_{7},\;N_{cn}\right)\right) \end{array}$$

(31) $$\begin{array}{l} \frac{dG_{3}}{dt}=\frac{1}{\tau_{6}}\left(-G_{3}+\text{Ψ}\left(B_{cn},\;G_{1},\;M_{cn}\right)\right) \end{array}$$

(32) $$\begin{array}{l} \frac{dG_{4}}{dt}=\frac{1}{\tau_{6}}\left(-G_{4}+\text{Ψ}\left(B_{cn},\;G_{2},\;M_{cn}\right)\right) \end{array}$$

(33) $$\begin{array}{l} \frac{dG_{5}}{dt}=\frac{1}{\tau_{6}}\left(-G_{5}+\frac{A_{cn}}{1+e^{-\Theta \left(gG_{1}-hG_{2}\right)}}\right) \end{array}$$

(34) $$\begin{array}{l} \frac{dG_{6}}{dt}=\frac{1}{\tau_{6}}\left(-G_{6}+\text{Ψ}\left(A_{cn},\;G_{1},\;N_{cn}\right)\right) \end{array}$$

(35) $$\begin{array}{l} \frac{dG_{7}}{dt}=\frac{1}{\tau_{6}}\left(-G_{7}+\text{Ψ}\left(A_{cn},\;G_{2},\;N_{cn}\right)\right) \end{array}$$

To feed the meta-control circuit, first, the information of the novelty *S*_1_ is extended for a longer time by entering a short-term memory circuit. By forced complementarity, the signal *S*_2_ is obtained. This signal together with *X*_1_ enters the control network that modulates the appropriate behaviors in the nonlinear oscillation-generating circuits, whose outputs are shown in Figure 13.

These circuits take inspiration from studies of the amygdala and cingulate cortex *via* Pontine Tegmental Nucleus (PTN) (Ramirez-Moreno and Sejnowski, 2012). Without them, the automaton, like its biological counterpart, in this case, would not be able to differentiate either extreme risk or security.

#### 2.2.7. Non-linear oscillation circuits. Lateral inhibition

In CNS, the non-linear oscillation circuits are responsible for generating repetitive oscillating signals, presented in the execution of motor actions such as breathing, eating, and swimming, among others. In this work, the magnitude of these oscillations will be the speed level applied to each actuator of the differential robot.


(36) $$\begin{array}{l} \frac{dL_{n}}{dt}=\frac{1}{\tau_{7}}\left(-L_{n}+\text{Ψ}\left(A-\rho G,\;\lambda ,\;B+L_{\left(n+2\right)}\right)\right) \end{array}$$

(37) $$\begin{array}{l} \frac{dL_{i}}{dt}=\frac{1}{\tau_{8}}\left(-L_{i}+\beta L_{\left(i-2\right)}\right) \end{array}$$

(38) $$\begin{array}{l} \lambda =K_{j}f\left(O_{1}+A_{P}-A_{Q}\right)-dL_{\left(2-\left(n-1\right)\right)}+{\left(-1\right)}^{n+1}\psi A_{P} \end{array}$$

(39) $$\begin{array}{l} n\in \left\{1,2\right\},\;i\in \left\{3,4\right\},\;j\in \left\{1,2\right\} \end{array}$$

**Figure 8:**
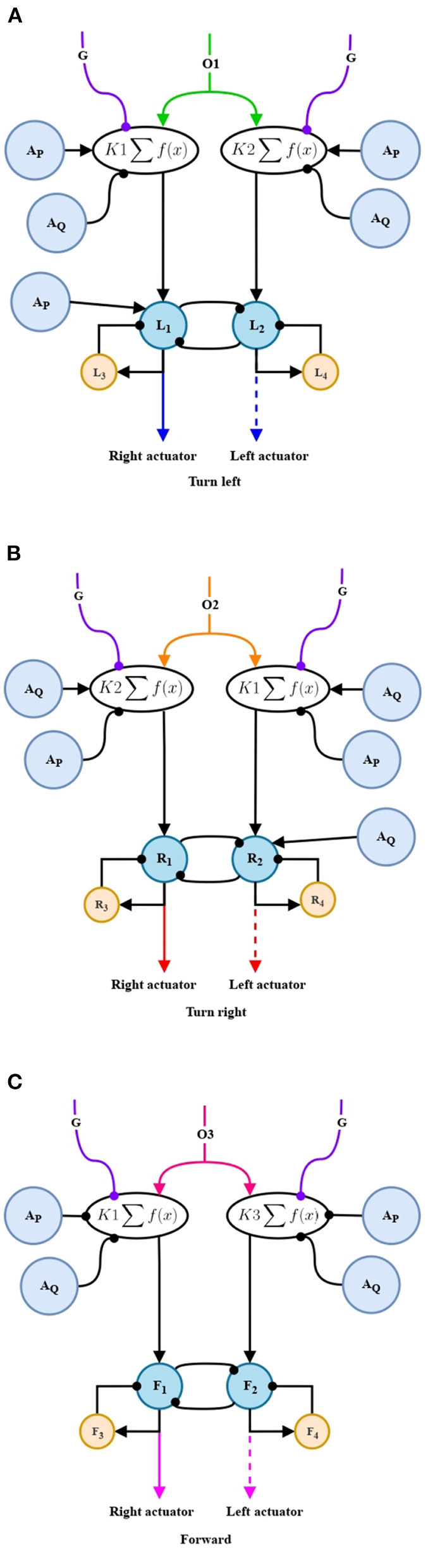
Non-linear oscillation generator circuit. **(A)** Non-linear oscillation circuit for turning left. **(B)** Non-linear oscillation generator circuit for turning right. **(C)** Non-linear oscillation generator circuit for forward motion.

In Figure 8A the *O*_1_ projection will be in charge of giving the order to generate the oscillations to perform a left-turn behavior. This will be fed to a parameter adaptation stage, note the order in which the connections coming from *A*_*P*_ and *A*_*Q*_ are given, these units refer to what is obtained in a stage of adaptation of left and right turning. Consequently, the left-turn adaptation (*A*_*P*_) presents an excitatory connection contrary to the inhibition of the right-turn adaptation (*A*_*Q*_). Our aim is to prolong this behavior over time. The projections of this stage continue to the nonlinear oscillation generator circuit. In this circuit, a left-turn adaptation unit is added again with the intention of increasing the difference between the widths of the oscillations and generate a torque that allows to change the orientation of the robot. The mathematical representation is given in Equations (36)–(39).


(40) $$\begin{array}{l} \frac{dR_{n}}{dt}=\frac{1}{\tau_{7}}\left(-R_{n}+\Psi (A-\rho G,\lambda ,B+R_{\left(n+2\right)}\right) \end{array}$$

(41) $$\begin{array}{l} \frac{dR_{i}}{dt}=\frac{1}{\tau_{8}}\left(-R_{i}+\beta R_{\left(i-2\right)}\right) \end{array}$$

(42) $$\begin{array}{l} \lambda =K_{j}f\left(O_{2}+A_{Q}-A_{P}\right)-dR_{\left(2-\left(n-1\right)\right)}+{\left(-1\right)}^{n}\psi A_{Q} \end{array}$$

(43) $$\begin{array}{l} n\in \left\{1,2\right\},\;i\in \left\{3,4\right\},\;j\in \left\{2,1\right\} \end{array}$$

For the generation of the right-turn swing oscillations (Figure 8B), the same structure and principle is used. However, care must be taken, once again, with the connections of the adaptation units. In this case, the right-turn adaptation (*A*_*Q*_) has excitatory connections, and the left-turn adaptation (*A*_*P*_) has inhibitory connections. Likewise, the right-turn adaptation unit is added to the oscillation generator circuit to generate the difference in the width of the oscillations, in this case in the opposite signal to that of the right-turn and, in that way, to rotate in the opposite direction. The mathematical representation is given in Equations (40)–(43).


(44) $$\begin{array}{l} \frac{dF_{n}}{dt}=\frac{1}{\tau_{7}}\left(-F_{n}+\text{Ψ}\left(A-\rho G,\;K_{\left(2j+1\right)}\lambda ,\;B+F_{\left(n+2\right)}\right)\right) \end{array}$$

(45) $$\begin{array}{l} \frac{dF_{i}}{dt}=\frac{1}{\tau_{8}}\left(-F_{i}+\beta F_{\left(i-2\right)}\right); \end{array}$$

(46) $$\begin{array}{l} \lambda =f\left(O_{3}-A_{P}-A_{Q}\right)-dF_{2-\left(n-1\right)} \end{array}$$

(47) $$\begin{array}{l} n\in \left\{1,2\right\},\;i\in \left\{3,4\right\},\;j\in \left\{0,1\right\} \end{array}$$

Finally, for the generation of oscillations corresponding to the forward motion (Figure 8C), both adaptations (*A*_*P*_ and *A*_*Q*_) project inhibitory connections, taking into account that in the established design the forward motion is expected to be less predominant. The mathematical representation is given in Equations (44)–(47).

### 2.3. Software configuration

For the virtual implementation, we made use of the robot *Burger* from the TurtleBot3 open source libraries (Open Source Robotics Foundation, 2020). The simulated environment was performed in the Gazebo simulator (Foundation, 2014). The middle-ware used was ROS (Robotics, 2021).

### 2.4. Hardware configuration

**Figure 9:**
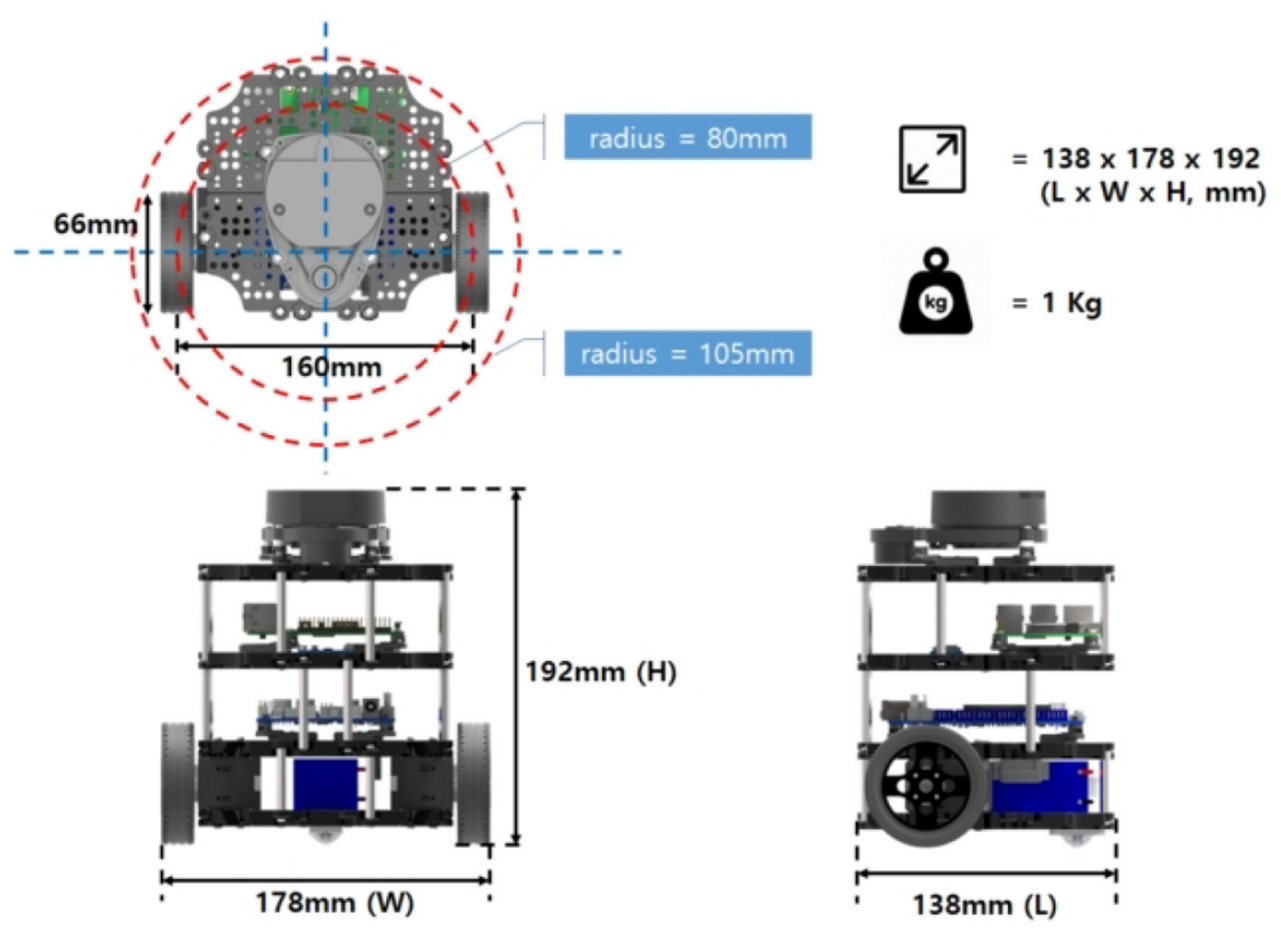
Turtlebot3 *Burger* model dimensions. Taken from Robotis (2022).

A TurtleBot3 *Burger* platform was used as the mobile automaton. This robot is configured with a 360-degree LDS-01 LiDAR sensor, a Raspberry Pi 3 Model B board for processing, and an OpenCR board for hardware control. The wheels actuator is the Dynamixel XL430-W250 motor. All the system is powered by a 3 cell LiPo battery of 11.1v and 2.2 Ah. The robot dimensions are visualized in Figure 9.

The robotic platform was configured with ROS Kinectic middleware installed on a Raspbian Buster operating system. The processing of the bio-inspired exploration system was tested both on the embedded and on an external computing unit, the latter configured with ROS noetic, Ubuntu 20.04, an Intel Icore i7 8th generation processor, and 16 GB ram memory. The communication between the embedded and the computational unit was done *via* WiFi.

### 2.5. Signal processing

**Figure 10:**
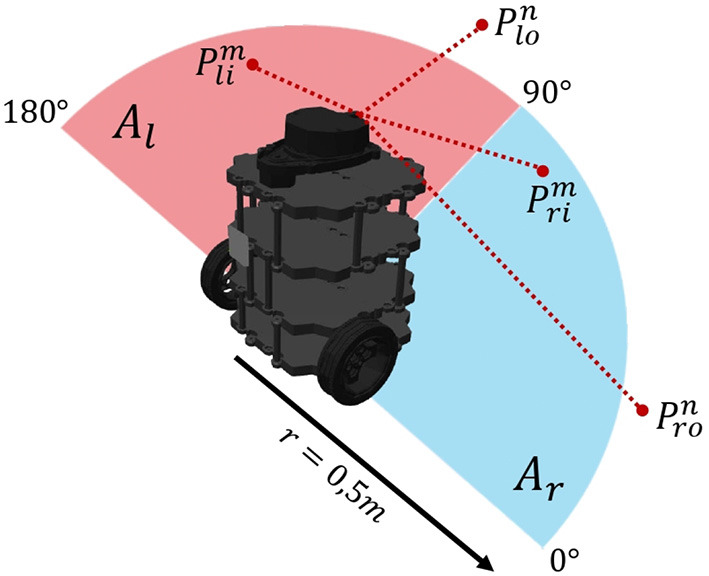
Signal processing. Take *l* for left and *r* for right. Take *i* for inside and *o* for outside.

Considering that the objective of the terrestrial navigation platform is to perform an obstacle avoidance exploration behavior, it was proposed to make use of the information captured by the LiDAR sensor to generate the input signals to the bio-inspired network. This was processed as shown in Figure 10. Just frontal information provided by the sensor was considered and was divided into two areas *A*_*r*_ (0°–90°) and *A*_*l*_ (90°–180°). A safety area of 0.5*m* radius was defined, with which it is defined that: points belonging to the degree range of the *A*_*r*_ area are classified into points inside the safety area (*P*_*r*_*i*__) and points outside the safety area (*P*_*r*_*o*__), likewise for the *P*_*l*_*i*__ and *P*_*l*_*o*__ points of *A*_*l*_. Points inside the safety area are penalized with a value of −1, while points outside the safety area are assigned with a value of +1. So, the values assigned to the areas *A*_*l*_ and *A*_*r*_ are defined as shown in Equations (48), (49). The former processing is intended to define in which direction (right or left) obstacles are closer to the robot so that the robot will head toward the clearest area.

As mentioned in Section 2.2.6, the aim of incorporating a basal ganglia-inspired meta-control network is to mediate the decisions made by the main network. It is proposed that the meta-control network will act on decisions where the robot's environment changes dramatically, for instance, when there is the presence of dynamic objects. To detect this, it is proposed to keep a record of the result obtained from the areas at instant *t* − 1 and compare it with those obtained at instant *t*. If a difference greater than a threshold ϵ exists, a value of 100 will be given to the signal *S*_1_ of the meta-control network in Equation (50) (Section 2.2.6).


(48) $$\begin{array}{l} A_{r}=\overset{90^{{}^{\circ}}}{\underset{k=0^{{}^{\circ}}}{\sum }}P_{r_{o}}^{k}+P_{r_{i}}^{k};\;P_{r_{o}}^{k}=+1,\;P_{r_{i}}^{k}=-1 \end{array}$$

(49) $$\begin{array}{l} A_{l}=\overset{180^{{}^{\circ}}}{\underset{k=90^{{}^{\circ}}}{\sum }}P_{l_{o}}^{k}+P_{l_{i}}^{k};\;P_{l_{i}}^{k}=-1,\;P_{l_{o}}^{k}=+1 \end{array}$$

(50) $$\begin{array}{l} S_{1}=\{\begin{array}{ll} 100, & \frac{\left|A_{r_{t-1}}-A_{r_{t}}\right|}{A_{r_{t-1}}}>\epsilon \;and\;\frac{\left|A_{l_{t-1}}-A_{l_{t}}\right|}{A_{l_{t-1}}}>\epsilon \\ 0, & \text{otherwise} \end{array} \end{array}$$

(51) $$\begin{array}{l} S_{2}=\gamma -S_{1} \end{array}$$

## 3. Results

In this section, the results obtained from both the simulation part and its implementation in the TurtleBot3 *Burger* robot are presented. The performance of the automaton in the exploration task and in the obstacle avoidance task was measured.

### 3.1. Simulation

#### 3.1.1. Exploration task

**Figure 11:**
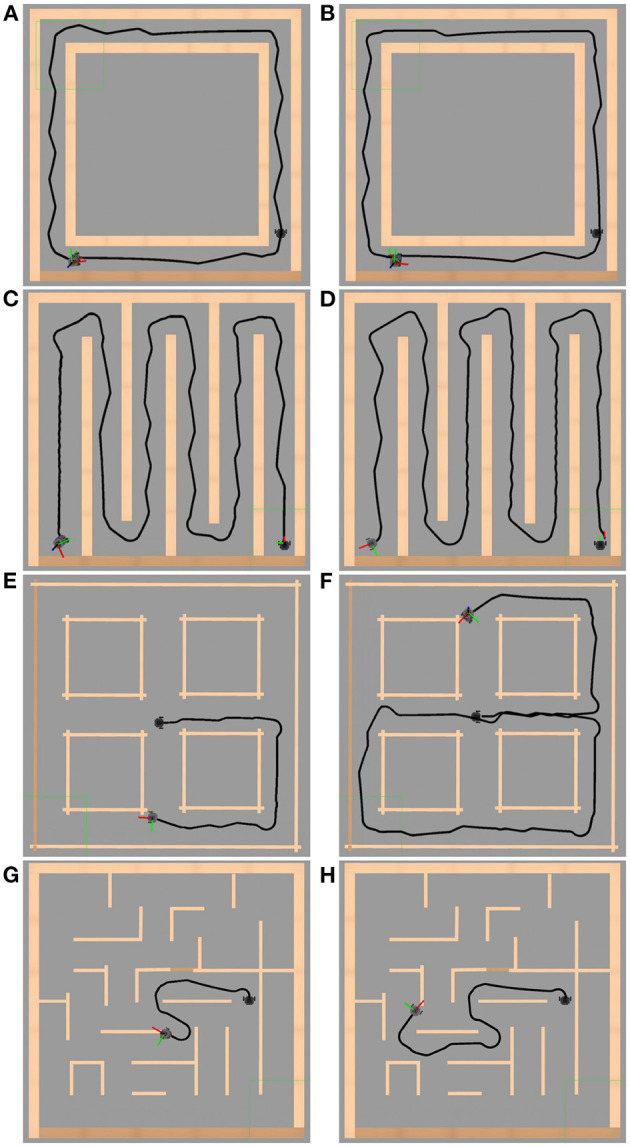
Simulation environment results. Figures on the left side show the Gazebo simulation environment without the meta-control circuit. The right side images show the trajectory made by the robot in the exploration behavior with the meta-control circuit. **(A, B)** Loop. **(C, D)** Zigzag. **(E, F)** Cross. **(G, H)** Traditional maze. In **(B, D, F, H)**, one can observe how we obtain a better performance using the meta-control network and allowing to achieve a greater trajectory in **(F, H)**.

To evaluate the performance of the exploration behavior, as well as its obstacle avoidance task, controlled by the bio-inspired neural network, the adaptation and simulation of the environments for exploration proposed in Yan et al. (2015) were implemented in Gazebo. In this work, the environments have a maximum exploration area of 4 *m*^2^. The maze walls are rigid and fully reflective surfaces, and the corridor width is, at least, 3 times the outside diameter of the robot. These mazes are denominated loop, cross, zigzag and traditional maze. In Figures 11A–D, the navigation in an established way is evaluated. In Figures 11E–H the environments are simulated until a collision or a deadlock situation takes place. The simulation results of these environments are presented in Figure 11.

Figure 11 illustrates the performance of the automaton without the meta-control network (left column, Figures 11A, C, E, G) and with the meta-control network (right column, Figures 11B, D, F, H). It is observed how the network modulates the right and left behaviors in the left column allowing better performance in the right column along the same path.

In the results shown in Figure 11, the mazes have a total area of 4.0 x 4.0 m with walls 1.0 m high and corridors 0.50 m wide. The environments in Figures 11A–D have 0.15 m wide walls, and the environments in Figures 11E–H have 0.05 m wide walls. The LiDAR sensor has a 360° field of view with a reading range of 0.12–3.50 m. Considering the safety area defined on the robot, Section 2.5, this field of view is reduced to 180° and a range of 0.12–0.50 m. In environments such as cross or traditional maze, if the width of its corridors is increased, it would cause the automaton to make a late decision between its three behaviors at interceptions, due to its actual change of vision, colliding with the outside corners while taking a wide-open curve. Considering the average speed of 0.04 m/s at which the automaton travels, this does not favor such decision-making. The opposite is true for loop and zigzag environments, where the automaton only decides between one of its behaviors.

### 3.2. Implementation

The bio-inspired neural network with neuromodulation designed in this work was mounted in the automaton TurtleBot3 *Burger* in order to measure its performance.

#### 3.2.1. Exploration task

**Figure 12:**
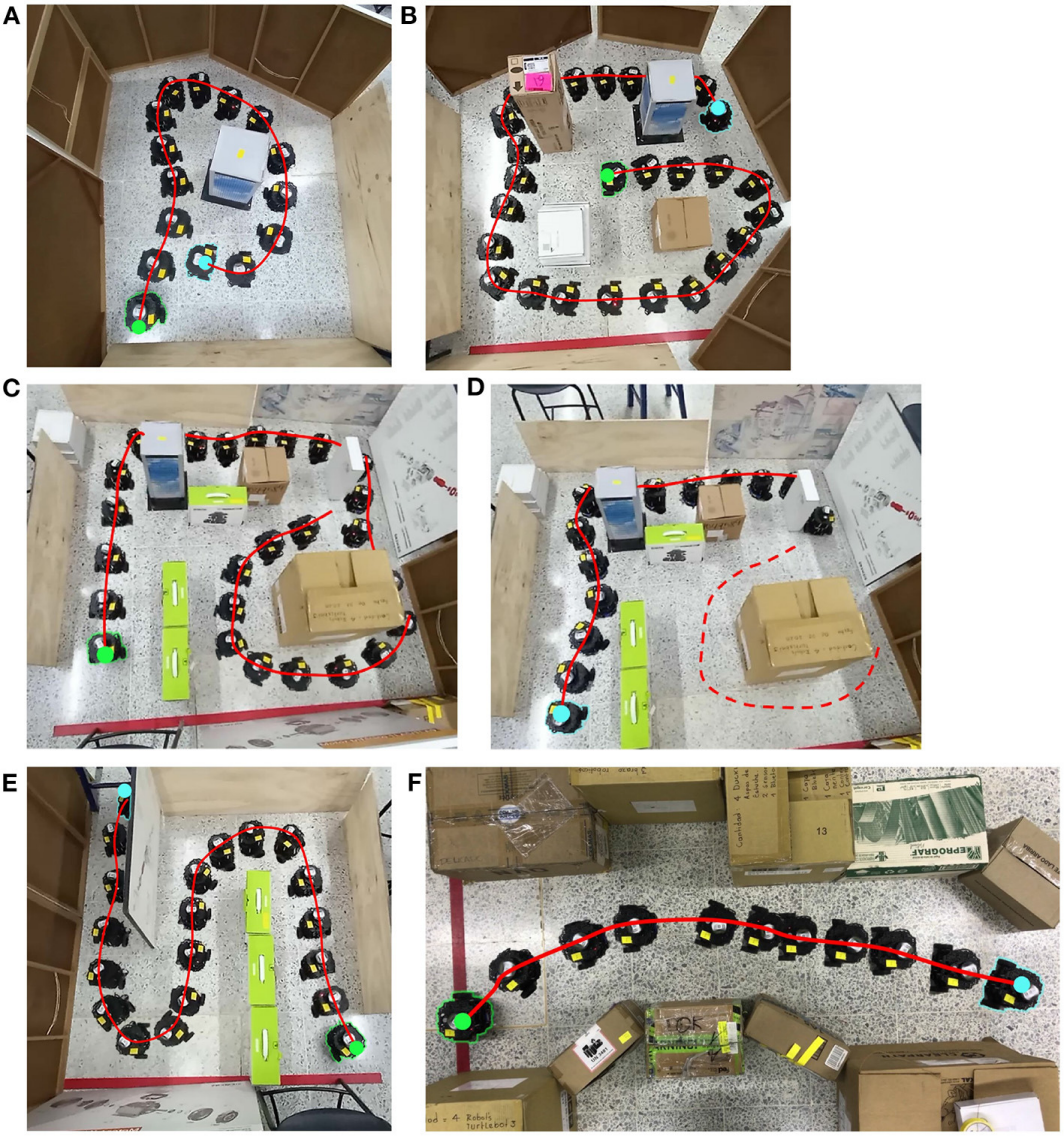
Implementation environment results. The path made by the automaton was drawn with red lines in each type of environment. Green circles are initial positions and blue circles are final positions. **(A)** Loop. **(B)** Cross. **(C, D)** Traditional maze part 1 and part 2, respectively. **(E)** Zigzag. **(F)** Simple maze. There one can be observed how the automaton completed the **(A, E, F)** environments successfully. In the **(B)** environment the automaton's performance started in the middle of the cross-environment and finished doing circles around the environment. In the **(C, D)** environments, there can be observed how the automaton's trajectory finishes at its starting point.

To evaluate the performance of the automaton in a natural environment, a hand-made maze was built, as shown in Figure 12. Each environment has a minimum of 1.0 *m*^2^ and a maximum of 2.0 *m*^2^; except for the simple maze that was built freely.

**Figure 13:**
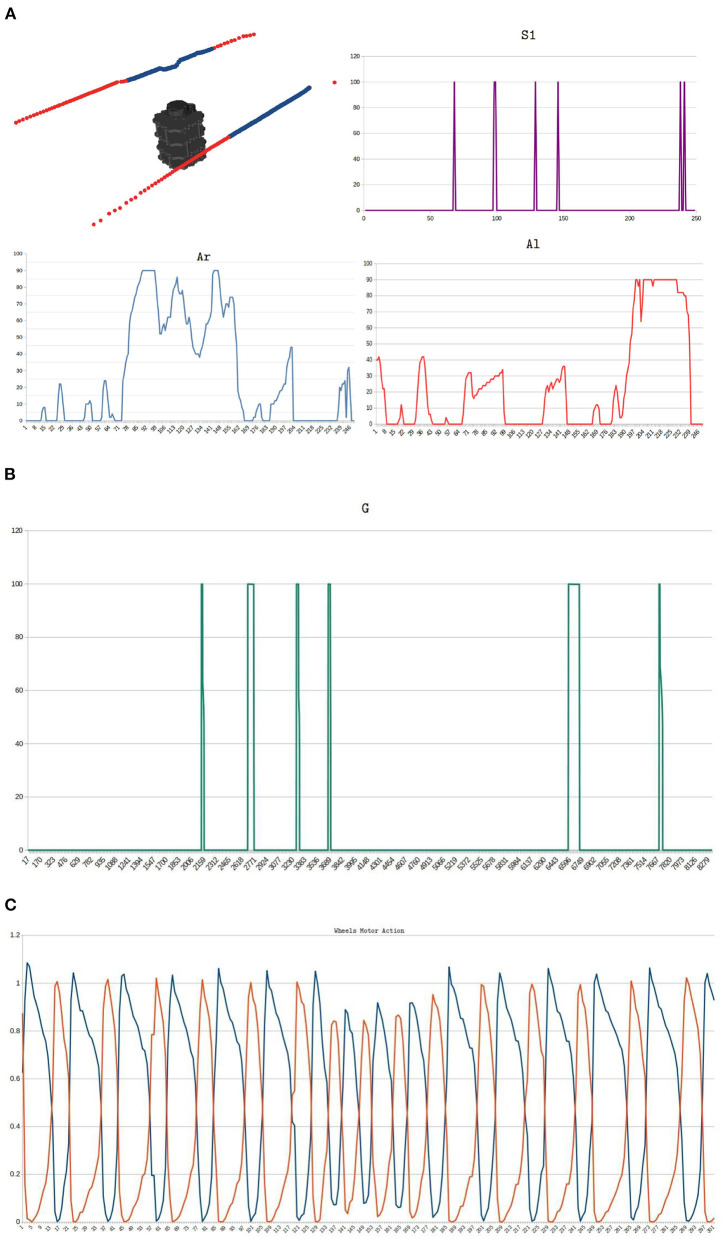
Implementation of the zigzag environment. **(A)** Real signals obtained from the environment and its processing in time. The top left image exhibits the LiDAR's points processing inside a corridor of the zigzag environment. Blue dots correspond to the points inside the safe area and red dots are the points outside the safe area. *S*_1_, *A*_*r*_, and *A*_*l*_ curves are the inputs signals for the bio-inspired network (Section 2.5), these were sampled within an interval of 360*ms*. The automaton's trajectory seen in Figure 12E is a result of processing *A*_*r*_ and *A*_*l*_ signals. The biggest values of *A*_*r*_ and *A*_*l*_ appear when the robot executes turns. **(B)** Meta-control circuit projection *G*. **(C)** Motor control signals of the mobile automaton. This signal was sampled within an interval of 1.0*ms*. Blue and orange signals correspond to the left wheel and the right wheel, respectively.

Figure 13 shows the signals obtained in the physical implementation of a zigzag environment. Figure 13A illustrates the information obtained from the real environment and its processing in time, top left image exhibits the LiDAR's points processing inside a corridor of the zigzag-maze, blue points correspond to points inside the safety zone and red points are those outside it. *S*_1_, *A*_*r*_, and *A*_*l*_ curves are the signals mentioned in Section 2.5, these were sampled within an interval of 360*ms*. Notice that *S*_1_ fires when there is an appropriate change in the values of *A*_*r*_ and *A*_*l*_ from one instant to another one. For instance, near sample 99 the *A*_*r*_ signal changes from 90 to 50 and *A*_*l*_ from 30 to 0, then, *S*_1_ triggers from 0 to 100. The automaton's trajectory seen in Figure 12E is a result of processing *A*_*r*_ and *A*_*l*_ signals. The biggest values of *A*_*r*_ and *A*_*l*_ appear when the robot executes turns. The projection of the meta-control network is shown in Figure 13B. Approaching sample 230 of *S*_1_, *A*_*r*_, and *A*_*l*_ signals, there were more obstacles inside the left area, thus, the robot must turn to the right. Figure 13C shows the wheels' motor action corresponds to this time, this signal was sampled within an interval of 1.0*ms*. The blue signal corresponds to the left wheel and the orange to the right. Blue oscillations are wider than orange oscillations, then, the left wheel spins more than the right wheel, and the right turn is made. When *S*_1_ fires, the modulation in the wheels' motor action is applied, and this generates a reduction in the amplitude of the oscillating signals. This reduces the automaton's velocity which gives time to taking a better decision.

#### 3.2.2. Meta-control circuit test

The performance of the neuromodulation network was tested by putting an obstacle (box) in the automaton's area vision. The automaton automatically avoids the obstacle and continues exploring (see Supplementary Videos 6, 8).

### 3.3. Metrics

To quantify the performance of the exploration in the different established environments the next metrics are proposed:

- Covered distance (*T*_*d*_): Covered distance by the robot measured in meters.
- Elapsed time (*T*_*t*_): Spent time in seconds.
- Average speed reached (*T*_*v*_): Quotient between *T*_*d*_ and *T*_*t*_.
- Exploration area (*E*_*a*_): Percentage of the total environment area covered by the robot.

These metrics values obtained for each simulated environment are presented in Table 2A.

**Table 2:** Metrics values for simulation environments in Figure 11.

**(A) Proposed metrics**								
**Proposed metrics**	**Loop**		**Cross**		**Zigzag**		**Traditional maze**	
	**W**	**W/O**	**W**	**W/O**	**W**	**W/O**	**W**	**W/O**
*T*_*d*_ [*m*]	13.4	13.3	15.25	5.25	22.76	22.1	4.48	2.94
*T*_*t*_ [*s*]	334	315	364	125	573	524	107	72
*T*_*v*_ [*m*/*s*]	0.040	0.042	0.042	0.042	0.039	0.042	0.042	0.040
*E*_*a*_ [%]	100	100	66.66	18.75	100	100	18.75	9.34
**(B) Error metrics for Loop and Cross maze**								
**Error metrics**	**Loop**				**Cross**			
	*x*		*y*		*x*		*y*	
	**W**	**W/O**	**W**	**W/O**	**W**	**W/O**	**W**	**W/O**
RMSE [*m*]	0.0194	0.0227	0.0324	0.0391	0.1906	0.2020	0.1904	0.2011
Mean error [*m*]	–0.0013	0.0018	0.0143	0.0185	0.0366	0.0403	–0.0483	-0.0515
Std error [*m*]	0.0193	0.0226	0.0291	0.0344	0.1871	0.1979	0.1842	0.1944
Max error [*m*]	0.0798	0.0839	0.0807	0.0807	1.610	1.610	0.1352	0.1352
Min error [*m*]	–0.0697	–0.0697	–0.0654	–0.0639	–0.0966	–0.0966	–1.613	–1.613
**(C) Error metrics for Zigzag and Traditional maze**								
**Error metrics**	**Zigzag**				**Traditional maze**			
	*x*		*y*		*x*		*y*	
	**W**	**W/O**	**W**	**W/O**	**W**	**W/O**	**W**	**W/O**
RMSE [*m*]	0.0298	0.0374	0.0268	0.0373	0.8881	0.9140	0.3396	0.3494
Mean error [*m*]	–0.0047	–0.0058	–0.0090	0.0025	–0.3256	–0.3452	0.0750	0.0787
Std error [*m*]	0.0294	0.0369	0.0253	0.0371	0.8263	0.8463	0.3312	0.3404
Max error [*m*]	0.1371	0.1709	0.1048	0.0955	1.682	1.682	1.413	1.414
Min error [*m*]	–0.1046	–0.1293	–0.1079	–0.1259	–1.857	–1.857	–1.252	–1.252

W refers to *With meta-control circuit* and W/O refers to *Without meta-control circuit*.

In order to compare quantitatively the performance evaluation of the automaton's trajectory and the optimum trajectory we added point-to-point metrics. The automaton's trajectory evaluation was evaluated considering the optimum trajectory, defined as the way that keeps in the middle of the corridors. In this comparison, the RMSE, mean error, standard deviation error, minimum error, and maximum error were computed for each axis. The results of the error metrics for each environment presented in Figure 11 are shown in Tables 2B, C.

## 4. Discussion and conclusion

The framework proposed in this work faces strong difficulties when it comes to navigate much more complex mazes (see Figures 11B, D). The automaton shows a very good performance in environments like those seen in Figures 11A, C. That difficulty is linked to the analysis of the environment information. Reducing the analysis to a specific area provoked a delay in the decision-making when an object appeared suddenly in front of the robot in open environments. Shortly, this problem could be solved by increasing the safety area, nevertheless, this could affect the performance in reduced space environments as shown in Figures 11A, C. The sensed area could be penalized with negatives. Due to this fact, it is proposed as part of future work the development and implementation of a bio-inspired strategy that allows a dynamic adjustment of the robot's safety area depending on the environment (wide or narrow areas).

The discussion presented above is also supported by the information presented in Table 2A. It shows the good performance exhibited by the cortical synaptic circuits adapted and applied, as mentioned in Section 2.2, for the exploration of unstructured environments in their entirety (*E*_*a*_). In addition, the performance of the automaton with and without meta-control network is shown in the error metrics in Tables 2B, C. The results illustrate that we obtain better performance with the implementation of this network. On average, the TurtleBot3 *Burger's* navigation speed was approximately 0.04*m*/*s*. By comparing with Miguel-Blanco and Manoonpong (2020) our exploration system is slow, similar to the one developed by Pardo-Cabrera et al. (2022).

A first approximation of the motor control of a mobile autonomous was proposed in Guerrero-Criollo et al. (2022). In that work, the input signals were simulated rather than being captured by a robust system. The meta-control network, which is responsible for detecting novelties, is also absent. In this work, we implement both the sensor part of the system that measures environmental data for inputs and the meta-control network. The bio-inspired network was implemented into the TurtleBot3 Burger embedded system. In this work, the design, simulation, and implementation of a bio-inspired neural network allows a differential robot to perform a safe exploration. An exploration task is defined as the behavior of traversing a terrain indefinitely while avoiding obstacles. Here, a framework is proposed to extract information from a LiDAR sensor that generates the input signals to the neural network online. Additionally, the implementation of a modulatory or meta-control network inspired by the basal ganglia is carried out. This network allows modulating the exploration behavior of the robot by reducing its speed progressively when drastic changes occur in the robot's environment within a safety area of 0.5*m* radius (see Figure 13D). As the robot advances through the mazes, this network detects novelties with greater priority, enabling it to avoid obstacles much more effectively. This was done with the aim of adding robustness to the bio-inspired exploration system against dynamic objects and reducing the reactivity of decision-making, thus improving the autonomy of the navigation system.

Frequently, to perform autonomous navigation tasks, it is required either that an operator previously walks with a registration system through the environment in which the robot will operate or that the operator teleoperates the robot while the registration of the area is being done. Either of the above two situations presents difficulties, the most obvious of which is the dependence on an operator in the robot's workflow for its operation. There are scenarios that can put the operator's safety at risks such as environmental disaster zones or mines. In addition, in these areas connectivity can be problematic to operate the robot remotely. For these reasons, it is considered that the proposed work can have a significant impact on exploration systems and the identification of unknown environments for ground platforms.

## Data availability statement

The original contributions presented in the study are included in the article/Supplementary material, further inquiries can be directed to the corresponding authors.

## Author contributions

RG-C, JC-L, and DR-M contributed to the writing of the manuscript. RG-C and JC-L proposed the architecture of the bio-inspired neural network and run the simulation and got the results. JH-L and DR-M proposed the neuromodulation network. All authors contributed to the article and approved the submitted version.
